# The use of antisera to epithelial membrane antigen in detecting micrometastases in histological sections.

**DOI:** 10.1038/bjc.1980.250

**Published:** 1980-09

**Authors:** J. P. Sloane, M. G. Ormerod, S. F. Imrie, R. C. Coombes

## Abstract

**Images:**


*Br. J. Cancer* (1980) **42**, 392

### THE USE OF ANTISERA TO EPITHELIAL MEMBRANE ANTIGEN IN DETECTING MICROMETASTASES IN HISTOLOGICAL SECTIONS

J. P. SLOANE*, M. G. ORMEROD†, S. F. IMRIE† AND R. C. COOMBES‡

*From the *Department of Histopathology, Royal Marsden Hospital, the †Division of Pathology, Institute of Cancer Research, and the ‡Ludwig Institute for Cancer Research, Downs Road, Sutton, Surrey*

Received 2 June 1980 Accepted 19 June 1980

**Summary.—We have investigated, by immunocytochemical means, the value of an antiserum raised to milk-fat-globule membranes in detecting metastatic deposits of breast carcinoma in conventional histological sections of liver, lymph nodes and marrow. The antiserum recognizes a membrane component, which we have called Epithelial Membrane Antigen, and which is confined to but widely distributed in epithelial tissues and tumours derived from them. In the sections examined, a greater extent of tumour infiltration was usually found, largely due to the identification of single malignant cells which normally go unrecognized with conventional stains. The number of positive samples was only increased, however, in sections of marrow aspirates, and the reasons for this are discussed. We suggest that further increases in detection rates could be attained by using the antiserum on cytological smears of marrow or even in cell suspensions.**

WE HAVE DESCRIBED the localization of a glycoprotein which we have called Epithelial Membrane Antigen (EMA), on neoplastic epithelial surfaces and luminal membranes in human tissue (Heyderman *et al.*, 1979; Sloane & Ormerod, 1980). The antigen was recognized by antisera raised to milk-fat-globule membranes, which are prepared by de-fatting human cream and are thought to be derived from the luminal membranes of lactating epithelial cells. It survives formalin fixation and paraffin embedding, so that it can be located in routine histological sections by standard immunohistochemical techniques. All major classes of epithelial tumours are capable of expressing EMA (Sloane & Ormerod, 1980). It is almost invariably localized on any lumina formed, but is also found frequently in the cytoplasm of the malignant cells. We have found that the cytoplasmic concentration of EMA was highest in cells occurring singly, without contact with other malignant cells, both in primary and secondary tumours (Sloane & Ormerod, 1980; Ormerod *et al.*, 1979). This effect allows the detection of micrometastases in tissues which are normally negative for EMA; such micrometastases might not be recognized by standard histological stains (Sloane & Ormerod, 1980; Ormerod *et al.*, 1979; Sloane *et al.*, 1980).

In this paper we investigate the value of immunohistochemical staining for EMA in detecting small metastases of mammary carcinoma in biopsies of liver, lymph nodes and marrow.

### MATERIALS AND METHODS

*Antisera.*—The antisera against EMA were obtained by injecting rabbits with defatted human cream suspended in complete Freund's adjuvant (Ceriani *et al.*, 1977; Heyderman *et al.*, 1979). The sera were absorbed with human plasma, extracts of liver and kidney, normal cross-reacting antigen, lactoferrin and a fraction from human milk (Heyderman *et al.*, 1979; Sloane & Ormerod, 1980).

The secondary antibodies were obtained by affinity-purification of a sheep anti-rabbit $\gamma$-globulin. They were conjugated either to horseradish peroxidase by Nakane's method (Nakane & Kawaoi, 1974) or to alkaline phosphatase using glutaraldehyde (Avrameas & Ternynck, 1971).

*Tissues*.—All tissues used were surgical or biopsy specimens removed from patients with breast cancer. With the exception of the marrow aspirates, tissues had been fixed in formol saline and embedded in paraffin-wax. Blocks of marrow aspirates were made after smears had been made for cytology. The material was fixed in Zenker's solution, embedded in 3% agar and then in paraffin. After dewaxing and before the further treatment described below, sections were treated with Lugol's iodine to remove mercury and then bleached in 5% sodium thiosulphate.

*Immunohistochemical stain*. — Approximately 4$\mu$m sections were dewaxed, bleached in a solution of hydrogen peroxide and treated with periodic acid, followed by borohydride (Heyderman & Neville, 1976). Sections were then incubated with a suitable dilution of rabbit antiserum for 90 min, washed and incubated with the enzyme-conjugated second antibody for a further hour and a half. The stain was developed in a solution of diaminobenzidine and hydrogen peroxide (for peroxidase) or naphthol AS:B1 phosphoric acid plus Brentamine Fast Red T.R. (for alkaline phosphatase). The counterstain was Mayer's haemalum.

The alkaline phosphatase conjugate was used on the sections of marrow because of the high level of endogenous peroxidase activity which was sometimes difficult to block adequately. Some batches of antiserum were found to contain an impurity antibody which reacted rather variably with erythrocytes. This had not been observed previously, because the antigen responsible was destroyed by treatment with periodate (used to destroy endogenous peroxidase). Therefore, the antiserum was absorbed further with red blood cells and, as a further precaution, all the sections were taken through periodate and borohydride.

As a negative control, in order to confirm the specificity of the staining, antiserum was absorbed with a preparation of EMA from human milk (Sloane & Ormerod, 1980; Steele & Ormerod, in preparation).

### PATIENTS

(a) *Liver biopsies*

These were obtained from 12 selected patients: 2 had scans showing liver metastases and the remainder showed abnormal uptake of isotope but no clear evidence of metastasis.

(b) *Lymph node biopsies*

Thirty-nine patients were studied, 23 with histologically negative nodes (8 of whom had developed metastases within 5 years) and 16 who had one or more positive nodes and distant metastases.

(c) *Marrow biopsies*

174 histological sections from 75 patients with breast cancer were examined with the immunohistochemical stain for EMA, and concomitantly the cytological smears were examined from these samples. In these aspirates, no malignant cells had been found on adjacent sections stained with haematoxylin and eosin (H. & E.).

The patients from whom the samples were obtained fell into 3 main groups:

(i) 20 patients who had no evidence of distant metastases were sampled at the time of mastectomy in 1975 and remain disease-free.
(ii) 8 patients were sampled at 7 sites (2 lumbar vertebrae and 5 samples of iliac crest) at the time of mastectomy. None of these had evidence of distant metastases but all had histologically involved axillary nodes, and therefore represent a subgroup with poor prognosis. None of these, however, have developed metastases 9–12 months since the date of sampling.
(iii) Iliac-crest marrow aspirates were examined on 73 separate occasions from 43 patients with metastatic breast cancer, specifically chosen since they had no evidence of marrow infiltration on H. & E.-stained sections. Sixteen out of 43 patients had skeletal deposits evident on either the bone scan or the radiological skeletal survey.

### RESULTS

(a) *Distribution of staining*

In the normal breast, EMA was located

FIG. 1.—Left: Normal human breast stained for EMA by the indirect immunoperoxidase technique. Right: Another section from the same block in which the antiserum has been absorbed with a purified preparation of EMA (×500).

FIG. 2.—Indirect immunoperoxidase stain of infiltrating ductal carcinoma of breast for EMA. Note luminal membrane staining, weaker cytoplasmic staining (with small intracytoplasmic vesicles) and patchy adjacent membrane staining (×500).

on the luminal membrane of the epithelial cells lining the ducts. There was often some weaker staining of the cytoplasm, but the adjacent cell membranes were consistently negative (Fig. 1). Myoepithelial cells and cells in the stroma were completely unstained. In mammary carcinomas, any lumina were almost invariably stained, while staining of the cytoplasm and the adjacent cell membranes was often present, sometimes patchily (Fig. 2). Larger metastases show similar features to primary tumours: heavy staining of lumina, with variable staining of cytoplasm and adjacent cell membranes. In minute deposits the marked feature was the large quantities of EMA in the cytoplasm of the cells. Fig. 3 shows an example of this in metastases found in the adrenal which was removed from a patient with disseminated breast carcinoma. Although there was sufficient tumour for positive identification from a section stained with H. & E., the immunohistochemical stain for EMA revealed additional cells not apparent in the H. & E. section. A similar effect has been seen in sections of liver, spleen and lymph nodes.

(b) *Liver metastases*

We extended this study by selecting 12 liver biopsies from patients with carcinoma of the breast (Table I). All but one of these biopsies had been reported negative on the basis of H. & E. staining. Staining for EMA did not reveal any positive cells in the 11 negative cases.

(c) *Lymph nodes*

We also looked at ipsilateral axillary lymph nodes removed from 31 patients at mastectomy for primary breast cancer (Table I). The results of the stain for EMA exactly paralleled the results with conventional histology. All recognizable deposits of carcinoma cells were positive for EMA. In no case did we pick up positive cells in nodes which had previously appeared negative.

FIG. 3.—Left: Adrenal containing metastatic breast carcinoma, stained for EMA. Right: Adjacent section stained with H. & E. (× 130).

TABLE I.—*Visualization of metastases in samples from breast-cancer patients*

| Site of biopsy | No. of patients | No. of patients with metastases | | | No. of samples examined | No. of samples with metastases detected by: | | |
|---|---|---|---|---|---|---|---|---|
| | | Nodal | Skeletal | Other | | H. & E. | EMA | Cytology |
| Liver | 12 | 12 | 0 | 2 | 12 | 1 | 1 | – |
| Lymph nodes | 23 | 0 | 0 | 0 | 292 | 0 | 0 | – |
| | 16 | 16 | 0 | 0 | 253 | 80 | 80 | – |
| Marrow (i) | 20 | 0 | 0 | 0 | 20 | 0 | 0 | 0 |
| (ii) | 8 | 8 | 0 | 0 | 28 | 0 | 0 | 0 |
| (iii) | 43 | 43 | 16 | 27 | 73 | 0 | 9 | 6 |

(d) *Marrow*

Twenty aspirates from patients presenting with primary breast cancer 3–5 years ago were studied. No patient has subsequently developed metastatic disease. Sections stained for EMA were all negative (Table I).

Eight patients with breast cancer of poor prognosis (axillary node involvement) were also studied. We examined sections of 1–5 aspirates from each patient, giving a total of 28 sections. All were negative.

The third series consisted of 73 blocks of marrow aspirates (see Table I), which were selected because malignant cells had not been found on the H. & E. sections, even though all 43 patients from whom they were taken had evidence of metastatic disease. All these patients had been staged as previously documented (Coombes *et al.*, 1980*a*,*b*). Thirty-one of the samples were from patients who had some evidence of bone involvement at the time the marrow was taken and the other 42 were from patients who were negative

FIG. 4.—Section of marrow aspirate containing single malignant cells stained for EMA by the indirect method using an alkaline phosphatase conjugate (× 500).

at the time. Nine sections from 8 patients had $EMA^+$ cells. (An example of a positive specimen is shown in Fig. 4.)

All the aspirates were used to make smears before the remaining cells were fixed and embedded. A Giemsa stain of these smears had revealed metastatic cells in 6 samples, two of them taken on separate occasions from one patient. As shown in Table II, in only 2 cases were $EMA^+$ cells found in a histological section and malignant cells found in a smear. Also shown in Table II are the results of the bone scan and the skeletal survey carried out when the aspirates were taken. In only one patient (No. 11) were $EMA^+$ cells unaccompanied by other evidence of metastases. This patient developed bone metastases 18 months later.

TABLE II.—*Comparison of stain for EMA in sections of marrow with other tests for marrow infiltration*

| Patient No. | EMA | Cytology | Bone scan | Skeletal survey |
|---|---|---|---|---|
| 1(a) | − | + | + | + |
| 2 | − | + | − | − |
| 3 | − | + | − | − |
| 4 | − | + | − | − |
| 1(b) | + | + | + | − |
| 5 | + | + | + | − |
| 6 | + | − | + | + |
| 7 | + | − | + | + |
| 8 | + | − | + | + |
| 9 | + | − | − | + |
| 10(a) | + | − | − | + |
| 10(b) | + | − | − | + |
| 11 | + | − | − | − |

### DISCUSSION

The purpose of this study was to determine whether small numbers of metastatic cells from breast carcinomas could be detected by an immunocytochemical stain for EMA. Our previous studies have shown that EMA can be used to visualize metastases, and we have also demonstrated the capacity of antisera to EMA to localize malignant cells in pleural and peritoneal effusions from patients with a wide variety of carcinomas, but the sensitivity of the method has not been determined, nor has it been compared with other methods of detecting metastatic disease.

The method of detection assumes that the $EMA^+$ cells seen in marrow are derived from the breast carcinoma. This has not been conclusively proved, but the balance of evidence weighs heavily in its favour for 3 reasons: (1) we have not seen cells normally in marrow which contain stainable quantities of EMA; (2) a specific stain for EMA has always been associated with cells of epithelial or mesothelial origin and (3) the positive cells show cytological features of malignancy. That the presence of such cells has prognostic value is borne out by their occurrence only in patients who had, or who subsequently developed, metastatic disease. No such cells were identified in any of the patients who had remained disease-free for 5 years.

The presence of large quantities of EMA in the cytoplasm of small metastases from breast carcinomas makes it a very sensitive indicator of metastatic disease, especially in tissues which do not normally express the antigen. Fig. 3 shows that the extent of infiltration of the adrenal exceeds that seen in the H. & E. sections. This increased detection is largely due to the recognition of single malignant cells, which are virtually impossible to identify with conventional stains. Although single cells were seen in the sections of lymph node and liver examined in this study, they were never seen in the absence of larger clumps which can also be identified in conventional sections. Thus, the number of positive specimens was not increased, even though a greater extent of infiltration may have been detected. This, however, should not detract from the value of the stain when applied to selected, problem biopsies where cells of dubious significance may sometimes be observed.

The marrow was an exception in that single cells were seen in the absence of clumps, and the number of positive samples was subsequently increased. The reason for this is unlikely to be inherent differences in the properties of tumour cells in different sites, and probably results from the act of aspiration itself, which may disrupt cell aggregates.

Table I shows that although in Group (iii) of the marrow samples the detection rate was increased from 0 to 9 out of 73 histological sections by staining for EMA, 6 samples were also identified as positive by conventional haematological smears. However, in Table II, it can be seen that the overlap between the two methods was only 2 specimens and that 7 of the EMA$^+$ samples were negative on cytological examination. It is the practice in this hospital to use most of the aspirated marrow for cytological examination, and only what remains for paraffin sections. Histological samples are thus of a somewhat variable size and often small. Sampling problems are further compounded by taking for a section only 5 $\mu$m from this residual tissue. Thus, the greater detection rate of malignant cells in smears than in H. & E. paraffin-embedded sections, may be accounted for simply by sample volume. At present it seems likely that detection rates would be increased even further by combining cytological and immunohistochemical techniques. Another alternative, which is being pursued, would permit the examination of even larger samples by the use of an immunofluorescent probe to scan the whole sample by flow cytofluorometric techniques (Scheifforth *et al.*, 1979).

The authors are indebted to Professor A. M. Neville for his advice and encouragement and to Mrs Kate Steele for her excellent technical assistance. M. G. Ormerod was supported by a project grant from the Medical Research Council.

REFERENCES

AVRAMEAS, S. & TERNYNCK, T. (1971) Peroxidase labelled antibody and Fab conjugates with enhanced intracellular penetration. *Immunochemistry*, **8**, 1175.

CERIANI, R. L., THOMPSON, K., PETERSON, J. A. & ABRAHAM, S. (1977) Surface differentiation antigens of human mammary epithelial cells carried on the human milk fat globule. *Proc. Natl Acad. Sci. U.S.A.*, **74**, 582.

COOMBES, R. C., POWLES, T. J., GAZET, J.-C., FORD, H. T., MCKINNA, A. & NEVILLE, A. M. (1980*a*) Assessment of biochemical tests to screen for metastases in patients with breast cancer. *Lancet*, i, 296.

COOMBES, R. C., POWLES, T. J., ABBOTT, M. & 4 others (1980*b*) Staging of human breast carcinoma. *Proc. R. Soc. Med.*, (in press).

HEYDERMAN, E. & NEVILLE, A. M. (1976) A shorter immunoperoxidase technique for the demonstration of carcinoembryonic antigen and other cell products. *J. Clin. Pathol.*, **30**, 138.

HEYDERMAN, E., STEELE, K. & ORMEROD, M. G. (1979) A new antigen on the epithelial membrane: Its immunoperoxidase localisation in normal and neoplastic tissue. *J. Clin. Pathol.*, **32**, 35.

NAKANE, P. K. & KAWAOI, A. (1974) Peroxidase labelled antibody: A new method of conjugation. *J. Histochem. Cytochem.*, **22**, 1084.

O'BRIEN, M. J., KIRKHAM, S. E., BURKE, B. & 4 others (1980) CEA, ZGM and EMA localisation in cells of pleural and peritoneal effusions. A preliminary study. *Invest. Cell Pathol.*, (in press).

ORMEROD, M. G., SLOANE, J. P. & STEELE, K. (1979) The localisation of epithelial membrane antigen in human mammary carcinoma by immunoperoxidase. *Proc. R. Micro. Soc.*, **14**, 243.

SCHEIFFORTH, O. F., VALET, G., DVORAK, R. & 4 others (1979) Flow cytometric characterisation of tumour associated changes in gynecologic malignancies. In *Separation of Cells and Sub-cellular Elements*. Ed. Peeters. Oxford: Pergamon Press. p. 11.

SLOANE, J. P. & ORMEROD, M. G. (1980) Distribution of epithelial membrane antigen in normal and neoplastic tissues and its value in diagnostic tumour pathology. *Cancer* (in press).

SLOANE, J. P., ORMEROD, M. G. & NEVILLE, A. M. (1980) Potential pathological application of immunocytochemical methods to the detection of micrometastases. *Cancer Res.*, **40**, 3079.


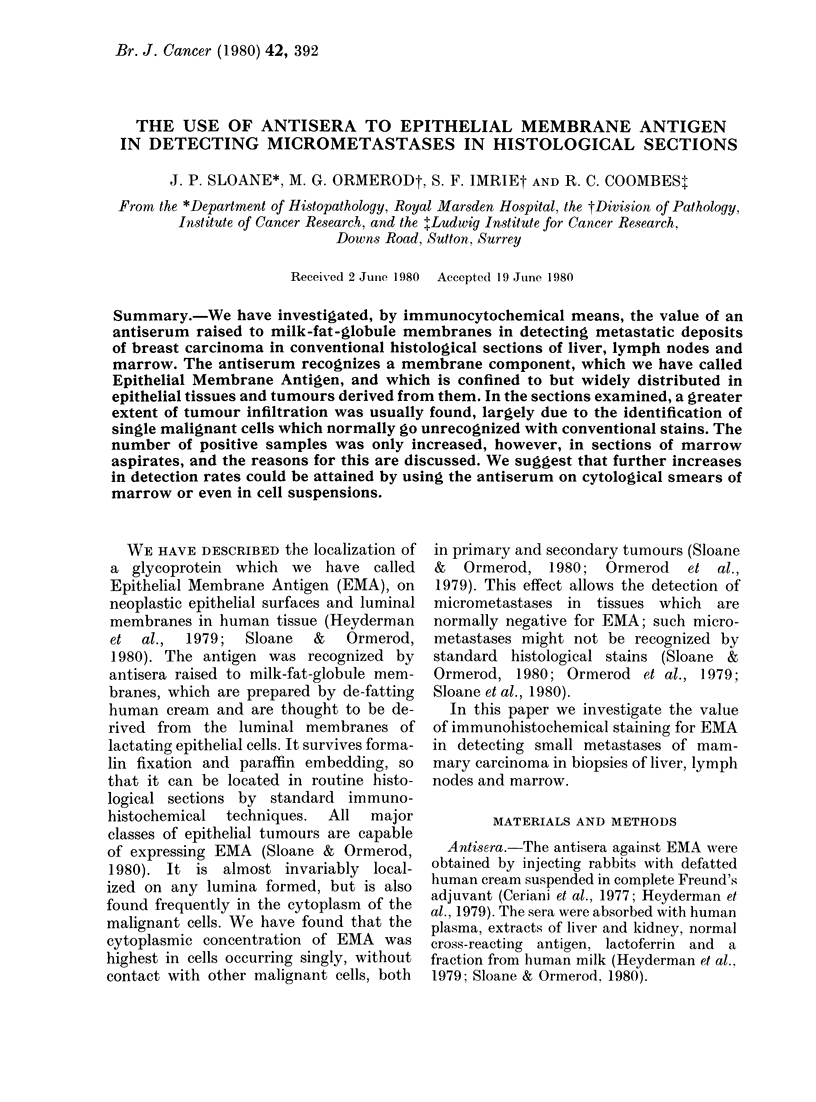

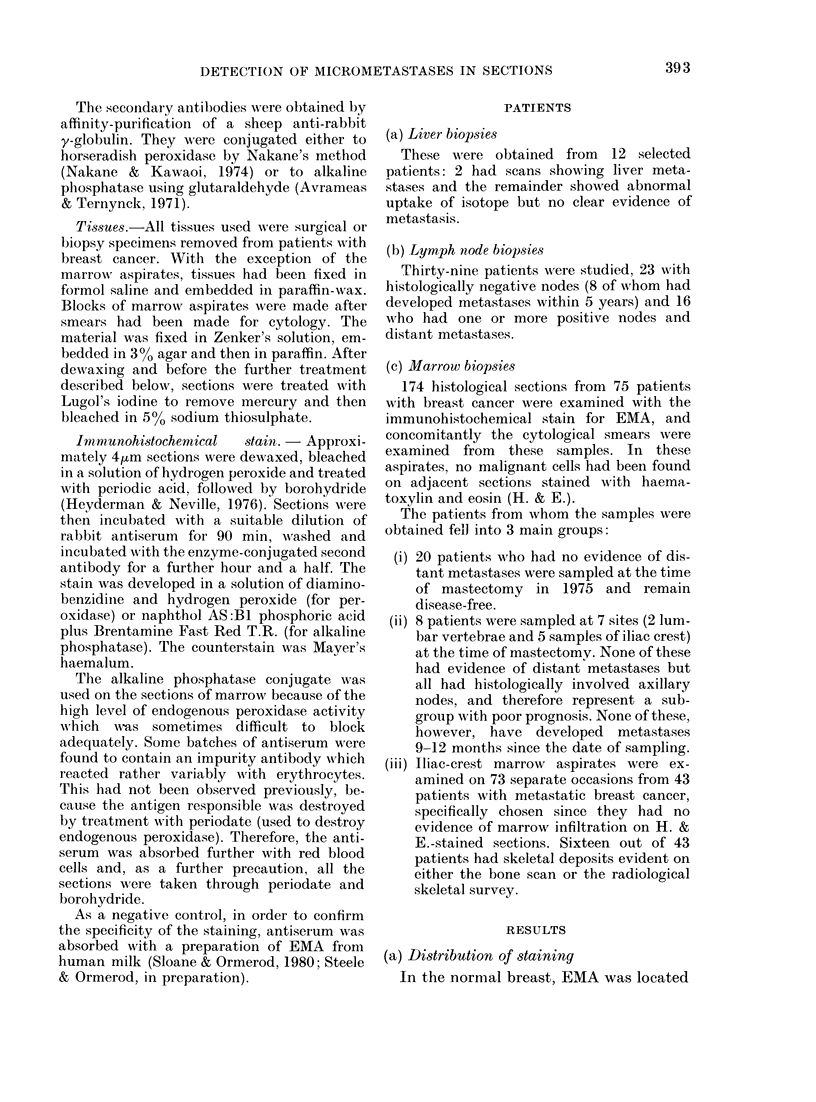

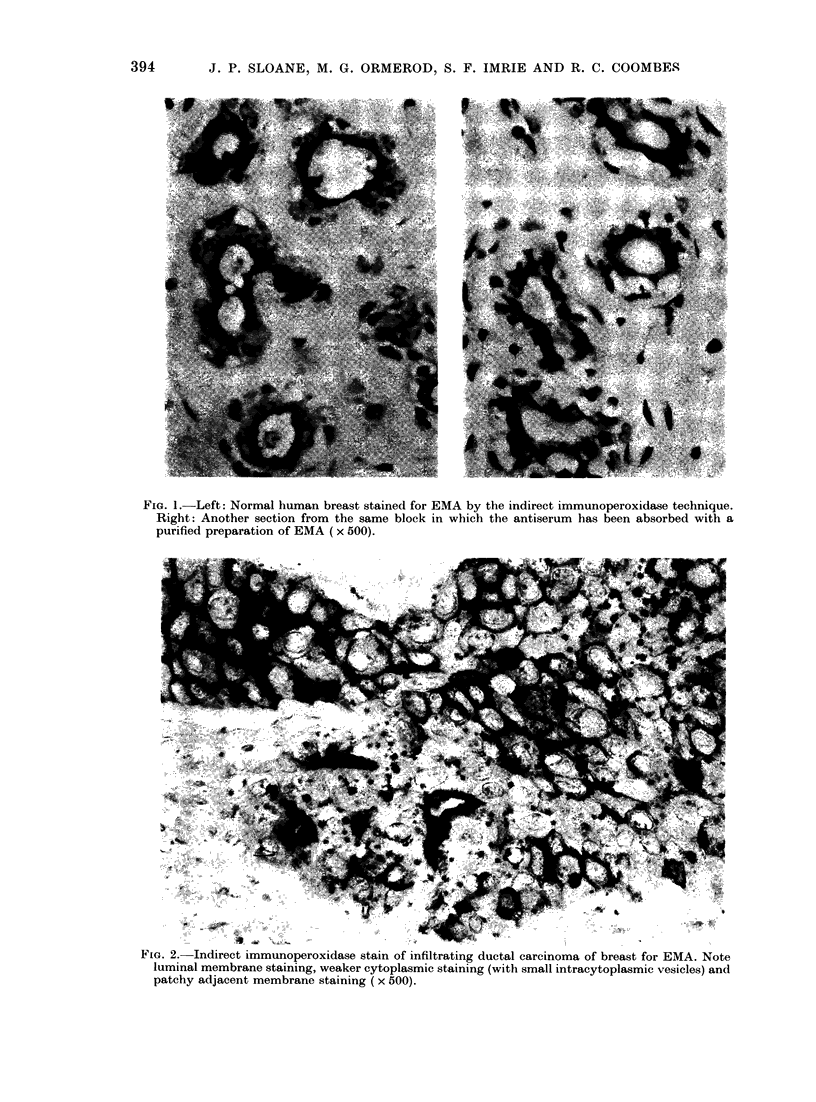

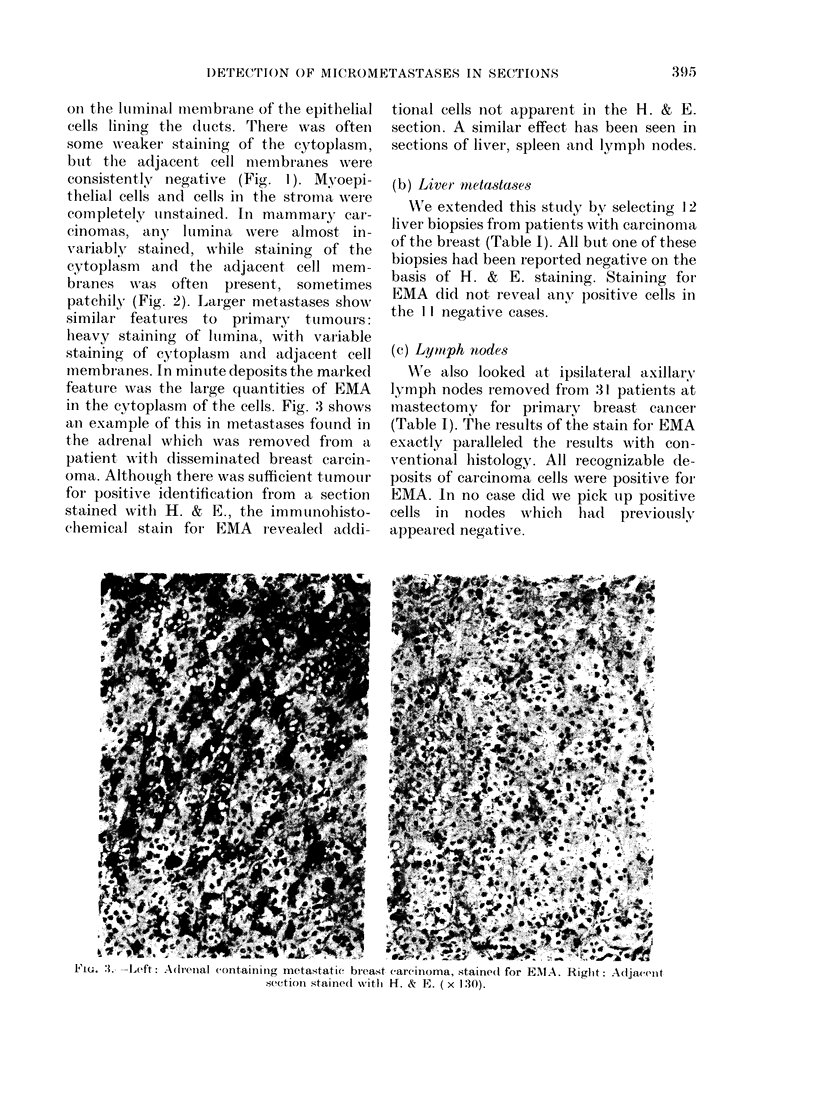

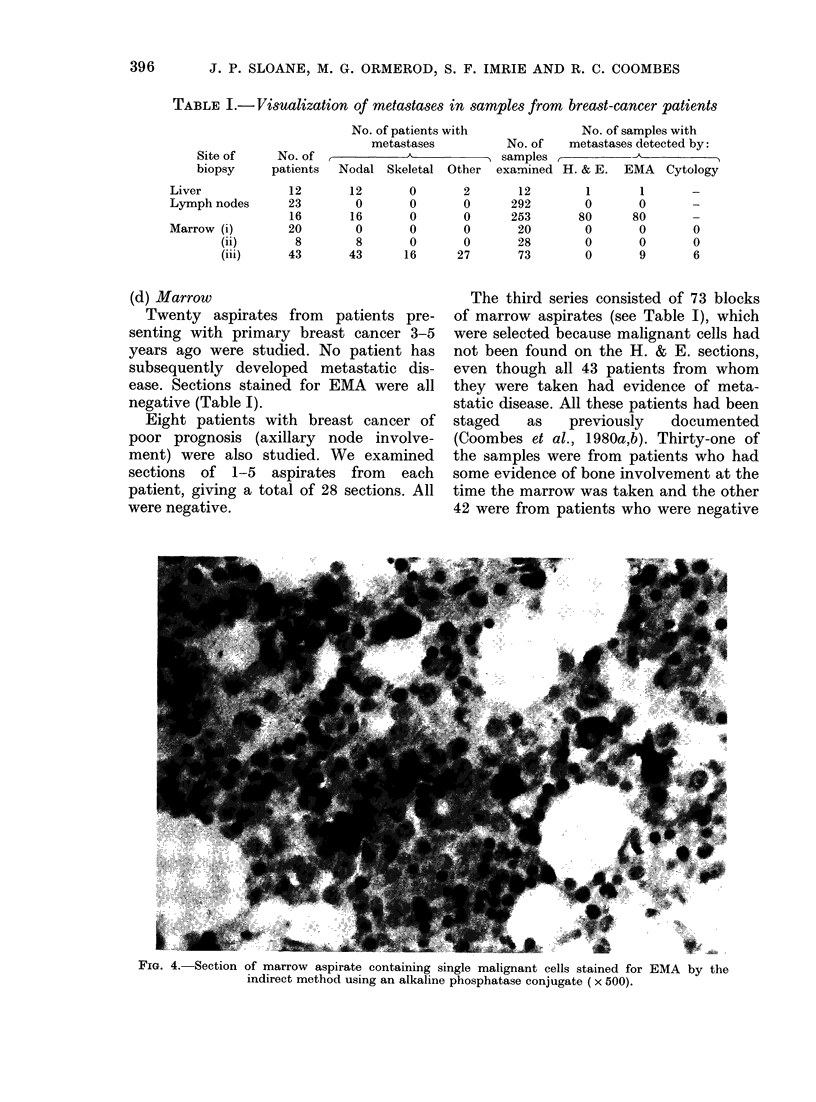

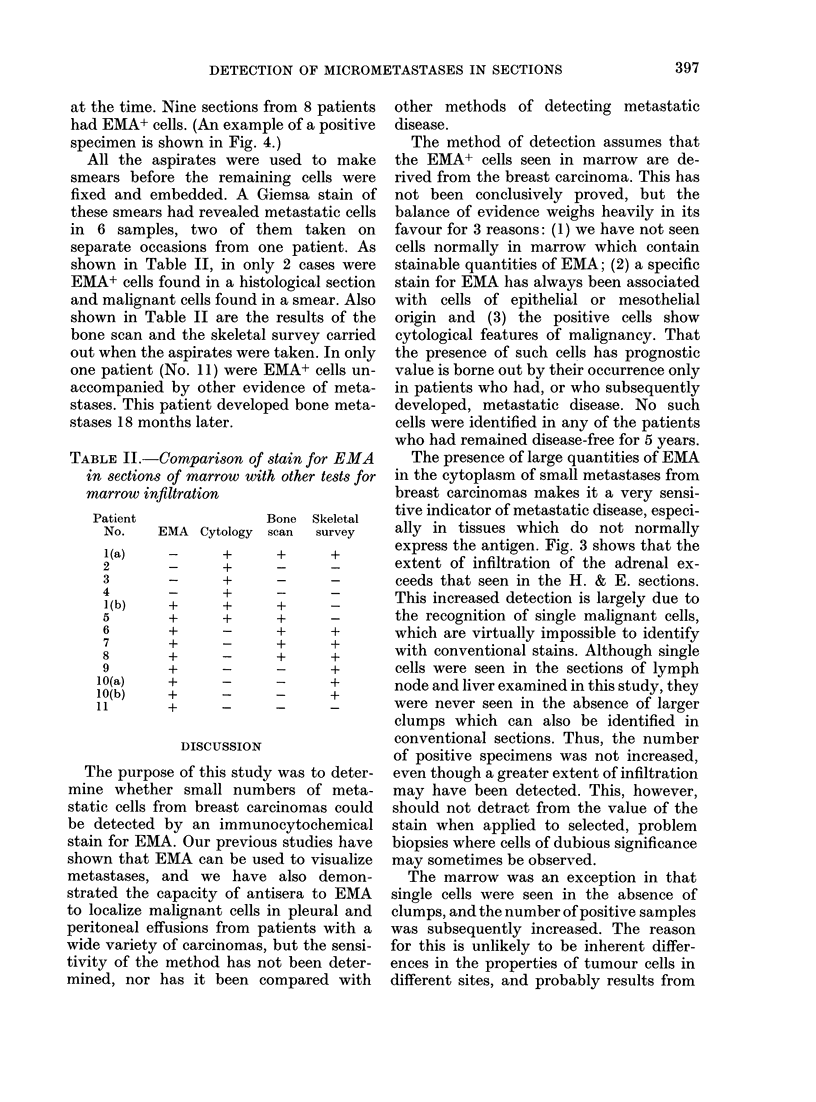

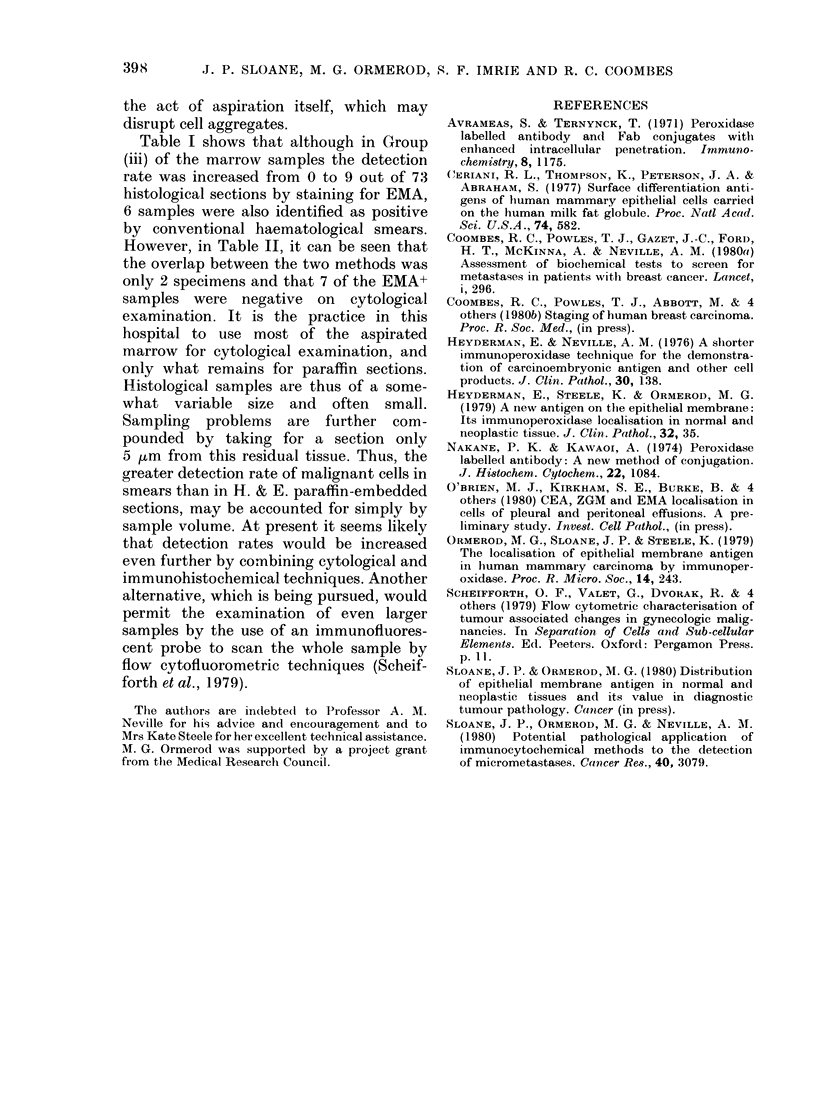


## References

[OCR_00607] Avrameas S., Ternynck T. (1971). Peroxidase labelled antibody and Fab conjugates with enhanced intracellular penetration.. Immunochemistry.

[OCR_00615] Ceriani R. L., Thompson K., Peterson J. A., Abraham S. (1977). Surface differentiation antigens of human mammary epithelial cells carried on the human milk fat globule.. Proc Natl Acad Sci U S A.

[OCR_00620] Coombes R. C., Powles T. J., Gazet J. C., Nash A. G., Ford H. T., McKinna A., Neville A. M. (1980). Assessment of biochemical tests to screen for metastases in patients with breast cancer.. Lancet.

[OCR_00632] Heyderman E., Neville A. M. (1977). A shorter immunoperoxidase technique for the demonstration of carcinoembryonic antigen and other cell products.. J Clin Pathol.

[OCR_00638] Heyderman E., Steele K., Ormerod M. G. (1979). A new antigen on the epithelial membrane: its immunoperoxidase localisation in normal and neoplastic tissue.. J Clin Pathol.

[OCR_00644] Nakane P. K., Kawaoi A. (1974). Peroxidase-labeled antibody. A new method of conjugation.. J Histochem Cytochem.

[OCR_00675] Sloane J. P., Ormerod M. G., Neville A. M. (1980). Potential pathological application of immunocytochemical methods to the detection of micrometastases.. Cancer Res.

